# Efficacy of Bevacizumab Combined with Pemetrexed in the Treatment of Recurrent and Metastatic Cervical Cancer

**DOI:** 10.3389/fsurg.2022.908101

**Published:** 2022-05-16

**Authors:** Ying He, Jing Wang, Shuangshuang Xie, Qianlong Xue

**Affiliations:** ^1^ Department of Gynecology, The First Affiliated Hospital of Hebei North University, Zhangjiakou, China; ^2^ Department of Emergency, The First Affiliated Hospital of Hebei North University, Zhangjiakou, China

**Keywords:** bevacizumab, pemetrexed, cervical cancer, efficacy, treatment

## Abstract

**Background:**

To investigate the efficacy and safety of bevacizumab combined with pemetrexed in the treatment of recurrent and metastatic cervical cancer.

**Methods:**

Clinical data of 65 patients with recurrent and metastatic cervical cancer who were admitted to our hospital were collected for retrospective analysis. All patients were administered with bevacizumab combined with pemetrexed for 4–6 cycles (21 days as 1 cycle). The short-term clinical efficacy and adverse reactions were compared between the two groups. In addition, the survival status of patients was followed up and recorded.

**Results:**

At least 4 cycles of chemotherapy were given to the 65 patients. There were 0 cases of complete response (CR), 14 cases of partial response (PR), 36 cases of stable disease (SD) and 15 cases of progressive disease (PD). The objective response rate (ORR) and the disease control rate (DCR) were 21.5% (14/65) and 76.9% (50/65), respectively. DCR was superior in patients with squamous cell carcinoma to that in those with adenocarcinoma (*p *= 0.039), but no statistically significant difference was found in ORR. Patients with extra-pelvic metastatic lesions had a better efficacy than those with intra-pelvic metastatic lesions, but the difference was not statistically significant (*p *> 0.05). The post-treatment adverse reactions mainly involved fatigue, nausea and vomiting, bleeding, leukopenia, anemia, thrombocytopenia, transaminase elevation, hypertension, proteinuria and neurotoxicity, most of which were grade I–II that ameliorated after symptomatic therapy. Grade III adverse reactions mainly included pain in 5 cases (7.7%), leukopenia in 17 cases (26.2%), anemia in 22 cases (33.8%), thrombocytopenia in 6 cases (9.2%), hypertension in 5 cases (7.7%) and neurotoxicity in 7 cases (10.8%). The follow-up results manifested that median overall survival (OS) and median progression-free survival (PFS) were 10.6 months and 6.6 months, respectively.

**Conclusion:**

Bevacizumab combined with pemetrexed exhibits certain efficacy in the treatment of recurrent and metastatic cervical cancer, with tolerable adverse reactions. Therefore, this therapeutic option deserves clinical popularization and application.

## Introduction

Patients with recurrent and metastatic cervical cancer are usually treated by means of chemotherapy, surgery and/or radiotherapy, but they have a poor prognosis. Generally, replacement of chemotherapy agents or optimal supportive therapy is available for those with failure of first-line chemotherapy. However, the therapeutic methods are reported to be limited (1). Bevacizumab can prolong the survival time of patients with advanced cervical cancer, as previously discussed, and has been approved by the European Union (EU) and U.S. Food and Drug Administration for the treatment of advanced cervical cancer (2, 3). According to National Comprehensive Cancer Network (NCCN) guidelines and expert consensus of the Chinese Society of Clinical Oncology (CSCO), pemetrexed, with favorable efficacy in the treatment of multiple tumors, is recommended for the second-line and above chemotherapy of cervical cancer (4, 5). This study aimed to investigate the efficacy and safety of bevacizumab combined with pemetrexed as a second-line chemotherapy regimen for patients with recurrent and metastatic cervical cancer.

## Materials and Methods

### General Data

Clinical data of 65 patients with recurrent and metastatic cervical cancer who were admitted to our hospital were collected. The patients were aged 36.4–71.1 years old, averagely (60.4 ± 9.4) years old. The inclusion criteria were as follows: (a) patients histopathologically diagnosed with cervical cancer, (b) those with disease progression or relapse after first-line combination chemotherapy or concurrent chemotherapy and radiotherapy, (c) those with a measurable target lesion on CT, MRI or ultrasound images, (d) those with basically normal routine blood tests and no obvious hepatic and renal dysfunction, (e) those with an Eastern Cooperative Oncology Group (ECOG) score of 0–2 points, and (f) those with an expected survival time ≥3 months. The exclusion criteria involved: (a) patients with allergy to pemetrexed or bevacizumab, (b) those with severe cardiac, pulmonary, hepatic or renal impairment, (c) those with coagulation disorder within 2 weeks prior to treatment, (d) those with hepatitis B or HIV infection, (e) those with requirement of anticoagulant therapy for relevant diseases, (f) those with other malignancies, or g) those with mental disorders or unable to cooperate in treatment for other reasons. The general information and clinical baseline data of patients are displayed in Table 1. This study complied with the *Declaration of Helsinki*, and all patients signed written informed consent.

**Table 1:** Baseline demographic and clinical characteristics of the studied patients.

Parameters	*n*, % (*n* = 65)
Age (years)	
<50	43 (66.2%)
≥50	22 (33.8%)
Histology	
Squamous cell carcinoma	49 (75.4%)
Adenocarcinoma	16 (24.6%)
Metastatic sites	
Pelvic	24 (36.9%)
Extrapelvic	41 (63.1%)
ECOG	
0	3 (4.6%)
1	45 (69.2%)
2	17 (26.2%)
Courses of chemotherapy	
4	13 (20.0%)
5	33 (50.8%)
6	19 (29.2%)

*Notes: ECOG, Eastern Cooperative Oncology Group.*

### Therapeutic Methods

Bevacizumab (Shanghai Roche Pharmaceutical Co., Ltd., Shanghai, China) was administered by intravenous drip, 15 mg/kg, on the 1st day. The dosage would be adjusted in case of body mass change more or less than 10%. Pemetrexed (Shandong Qilu Pharmaceutical Co., Ltd) 500 mg/m^2^ was given by intravenous drip (>10 min), on the 2nd day. The treatment lasted for 4–6 cycles (21 days as 1 cycle). One week prior to administration with pemetrexed, patients took orally 400 μg of folic acid daily, and were intramuscularly injected with 1,000 μg of vitamin B_12_. Dexamethasone (4 mg) was given orally, twice daily, 1 day before, on the day of and 1 day after treatment with pemetrexed. Folic acid was discontinued 21 days after last administration with pemetrexed. Vitamin B_12_ was injected once every 3 cycles.

### Observational Indicators

Response Evaluation Criteria in Solid Tumors (RECIST) v1.1 was utilized to evaluate the clinical efficacy with following indicators: complete response (CR), partial response (PR), stable disease (SD) and progressive disease (PD). Objective response rate (ORR) = (CR + PR)/(total cases) × 100%, and disease control rate (DCR) = (CR + PR + SD)/(total cases) × 100%.

During the follow-up period, adverse reactions were recorded and evaluated according to the National Cancer Institute Common Terminology Criteria for Adverse Events (NCI-CTCAE) v4.0.

Through follow-up visits, progression-free survival (PFS) and overall survival (OS) were recorded. The period from enrollment to disease progression or death was regarded as PFS, while that from enrollment to death for any reason was defined as OS. The follow-up period ended in May 2021.

### Statistical Analysis

Statistical Product and Service Solutions (SPSS) 22.0 software (IBM, Armonk, NY, USA) was adopted for statistical analysis. Measurement data were expressed by mean ± standard deviation $(\bar{\chi }\pm s)$ and compared by two-sample *t*-test between two groups. Enumeration data were expressed by percentage (%), and compared by chi-square test between two groups. Kaplan-Meier method was employed to plot the survival curve. *P *< 0.05 was considered statistically significant.

## Results

### Comparison of Short-Term Clinical Efficacy

At least 4 cycles of chemotherapy were given to the 65 patients. There were 0 cases of CR, 14 cases of PR, 36 cases of SD and 15 cases of PD. ORR and DCR were 21.5% (14/65) and 76.9% (50/65), respectively (Table 2).

**Table 2:** Tumor response of the studied patients.

Parameters	*n*, % (*n* = 65)
Complete response (CR)	0 (0%)
Partial response (PR)	14 (21.5%)
Stable disease (SD)	36 (55.4%)
Progressive disease (PD)	15 (23.1%)
ORR (CR + PR)	14 (21.5%)
DCR (CR + PR + SD)	50 (76.9%)

*Notes: ORR, Objective response rate; DCR, Disease control rate.*

There were 0 cases of CR, 12 cases of PR, 29 cases of SD and 8 cases of PD among 49 patients with squamous cell carcinoma. ORR and DCR were 24.5% (12/49) and 83.7% (41/49), respectively. Among 16 patients with adenocarcinoma, there were 0 cases of CR, 2 cases of PR, 7 cases of SD and 7 cases of PD. ORR and DCR were 12.5% (2/16) and 56.3% (9/16), respectively. Among 24 patients with intra-pelvic metastasis, there were 0 cases of CR, 4 cases of PR, 12 cases of SD and 8 cases of PD. ORR and DCR were 16.7% (4/24) and 66.7% (16/24), respectively. Among 41 patients with extra-pelvic metastasis, there were 0 cases of CR, 10 cases of PR, 24 cases of SD and 7 cases of PD. ORR and DCR were 24.4% (10/41) and 82.9% (34/41), respectively. After combination treatment with bevacizumab and pemetrexed, DCR was superior in patients with squamous cell carcinoma to that in those with adenocarcinoma (*p *= 0.039), but no statistically significant difference was found in ORR. Patients with extra-pelvic metastatic lesions had a better efficacy than those with intra-pelvic metastatic lesions, but the difference was not statistically significant (*p *> 0.05).

### Incidence of Adverse Reactions

The post-treatment adverse reactions mainly involved fatigue, nausea and vomiting, bleeding, leukopenia, anemia, thrombocytopenia, transaminase elevation, hypertension, proteinuria and neurotoxicity, mostly in grade I–II, which were ameliorated after symptomatic therapy. No grade IV adverse reactions and no progressive diseases and deaths related to adverse reactions were observed in both groups. Grade III adverse reactions mainly included pain in 5 cases (7.7%), leukopenia in 17 cases (26.2%), anemia in 22 cases (33.8%), thrombocytopenia in 6 cases (9.2%), hypertension in 5 cases (7.7%) and neurotoxicity in 7 cases (10.8%). The adverse reactions regarding bevacizumab were mainly hypertension, proteinuria and bleeding (Table 3).

**Table 3:** Comparison of adverse reactions of the studied patients

	Grade I–IV (*n* = 65)	Grade III–IV (*n* = 65)
Pain	21 (32.3%)	5 (7.7%)
Fatigue	39 (60.0%)	0 (0%)
Nausea and vomiting	35 (53.8%)	0 (0%)
Diarrhea	9 (13.8%)	0 (0%)
Bleeding	20 (30.8%)	0 (0%)
Leukopenia	33 (50.8%)	17 (26.2%)
Anemia	36 (55.4%)	22 (33.8%)
Thrombocytopenia	15 (23.1%)	6 (9.2%)
Transaminase elevation	12 (18.5%)	0 (0%)
Hypertension	16 (24.6%)	5 (7.7%)
Proteinuria	8 (12.3%)	0 (0%)
Thromboembolism	1 (1.5%)	0 (0%)
Neurotoxicity	8 (13.3%)	7 (10.8%)
Skin diseases	14 (21.5%)	0 (0%)

### Postoperative Follow-up Visits of Patients in the Two Groups

**Figure 1:**
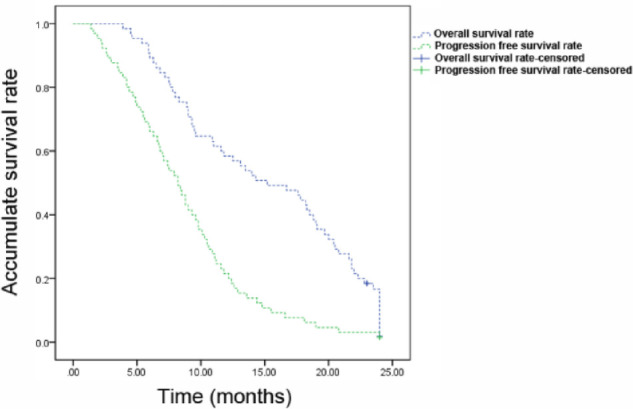
Kaplan-Meier survival curves of patients with recurrent metastatic cervical cancer. The overall survival rate and progression free survival rate of patients were shown.

As of May 2021, all patients were followed up for 3–24 months. The median OS and median PFS were 10.6 months and 6.6 months, respectively. Kaplan-Meier method was employed to plot the survival curve (Figure 1).

## Discussion

As a common malignant tumor affecting the female reproductive system, cervical cancer ranks second in the mortality rate among tumors in females (6). According to the statistics, about 130,000 cases are newly diagnosed with cervical cancer in China each year, and relapse and metastasis following treatment may occur in 15%–30% of patients. Limitations exist regarding therapeutic regimens for patients with refractory cervical cancer, especially those with continuous progression after multiple courses of radiotherapy and multi-line platinum-based combination chemotherapy. Then, platinum single-agent chemotherapy is usually administered again. However, the therapeutic efficacy is unsatisfactory, and the OS is short. Additionally, the 5-year survival rate is only 10%, and the quality of life is poor. For these reasons, numerous studies have been devoted to the targeted therapy which has become a trend of future development (7, 8).

Bevacizumab is a recombinant humanized monoclonal antibody that can selectively bind to human vascular endothelial growth factor (VEGF) and block its biological activity, and inhibit the binding of VEGF to its receptors Flt-1 and KDR on endothelial cells, thus reducing tumor angiogenesis and suppressing tumor growth (9). The extensive anti-tumor activity of bevacizumab has been reported among various cancers, such as colon cancer, non-small cell lung cancer, epithelial ovarian cancer and breast cancer (10–13). Moreover, bevacizumab is increasingly used in the treatment of cervical cancer. Wright et al. (14) reported that bevacizumab combined with 5-fluorouracil (5-FU) has a DCR reaching 67% and a median PFS of 4.3 months in the treatment of recurrent cervical cancer (14). The mid-term analysis of the U.S. GOG 240 manifested that after chemotherapy combined with bevacizumab, the survival period of patients who suffer from intractable, recurrent or metastatic cervical cancer has been prolonged by about 4 months, the median survival period is 17 months, the effective rate is 48%, and the mortality rate is reduced by 29% (3). Another multi-center study displayed a favorable progression-free survival after administration with bevacizumab, and exhibited survival period exceeding 6 months in 24% of patients, 7.3 months of median OS and a drug response rate of 10.9% (15). Therefore, bevacizumab has successively been approved by the EU and U.S. Food and drug administration for the treatment of advanced cervical cancer. Accordingly, cervical cancer has become the first gynecological tumor whose OS can be effectively prolonged by anti-angiogenesis agents (2). Furthermore, the latest version of NCCN guidelines has issued the application of bevacizumab combined with cisplatin and paclitaxel for recurrent or metastatic cervical cancer as the first-line therapeutic agents (16).

Pemetrexed is a synthetic multi-target antifolate and contains pyrrolidine grouping in structure, which can effectively inhibit dihydrofolate reductase, glycinamide ribonucleotide formyl transferase and thymidylate synthase and block thymidine and purine synthesis, thus affecting nucleic acid synthesis in tumor cells to exert anti-tumor effects (17). Previous studies have confirmed the effectiveness of single agent pemetrexed in the treatment of multiple tumors (18). The research on pemetrexed as a second-line treatment of cervical cancer manifested that 15% (4/27) and 59% (16/27) of patients achieve PR and SD, and PFS and OS are 3.1 months and 7.4 months, respectively (19). Pemetrexed was recommended for the second-line and above chemotherapy of cervical cancer by the NCCN guidelines in 2016.

In this study, ORR and DCR were 21.5% (14/65) and 76.9% (50/65), respectively, in patients with recurrent and metastatic cervical cancer receiving bevacizumab combined with pemetrexed as the second-line treatment. The follow-up results denoted that median OS and median PFS were 10.6 months and 6.6 months, respectively. DCR was superior in patients with squamous cell carcinoma to that in those with adenocarcinoma (*p *= 0.039), but no statistically significant difference was found in ORR. Patients with extra-pelvic metastatic lesions had a better efficacy than those with intra-pelvic metastatic lesions, but the difference was not statistically significant (*p *> 0.05). The findings demonstrated the certain efficacy of chemotherapy combined with targeted drugs. As reported, bevacizumab combined with chemotherapy exhibits better PR than pemetrexed or bevacizumab alone, greater SD than bevacizumab single agent but similar to pemetrexed, and more favorable PFS and OS than either of the two (15). Therefore, combination of bevacizumab and pemetrexed as a second-line treatment is superior to its single-agent therapy in terms of short-term clinical efficacy for patients with recurrent and metastatic cervical cancer. With respect to safety, this study revealed that the adverse reactions during treatment were mainly in grade I–II, and grade III–IV severe adverse reactions mainly included leukopenia, anemia, thrombocytopenia, hypertension and neurotoxicity, which could be basically tolerated. In addition, no death related to treatment was observed. These findings confirmed the safety of combined therapy.

The present study was a retrospective study with limited sample sizes and relatively short follow-up period, and the follow-up content was not comprehensive enough. Thus, in the future, multi-center, large-sample, prospective clinical trials should be carried out to validate the conclusions of this study.

## Conclusion

Bevacizumab combined with pemetrexed exhibits certain efficacy in the treatment of recurrent and metastatic cervical cancer, with tolerable adverse reactions. Therefore, this therapeutic option deserves clinical popularization and application.

## Data Availability

The original contributions presented in the study are included in the article/Supplementary Material, further inquiries can be directed to the corresponding author/s.
